# Predictors of 30-day outcomes following mitral valve repair

**DOI:** 10.1016/j.amsu.2019.09.001

**Published:** 2019-09-10

**Authors:** Adam M. Reisman, A. Taylor Thomas, Percy Boateng, I. Michael Leitman

**Affiliations:** a Departments of Medical Education, Icahn School of Medicine at Mount Sinai, United States; b Departments of Cardiovascular Surgery, Icahn School of Medicine at Mount Sinai, United States; c Departments of Surgery, Icahn School of Medicine at Mount Sinai, United States

**Keywords:** Cardiac surgery, Mitral valve, Mitral valve repair, Predictors, Outcomes

## Abstract

**Introduction:**

Mitral valve repair has been established as the preferred treatment option in the management of degenerative mitral valve disease. Compared with other surgical treatment options, mitral valve repair is associated with increased survival and decreased rates of both complications and reoperations. However, among patients undergoing mitral valve repair, little is known about the predictors of postoperative outcomes. The purpose of this study is to identify preoperative patient risk factors associated with postoperative morbidity and mortality within 30 days of mitral valve repair.

**Methods:**

Data was derived from the American College of Surgeons National Surgical Quality Improvement Program database to assess patients who underwent mitral valve repair from 2011 through 2017. Preoperative risk factors were analyzed to determine their association with a variety of postoperative 30-day outcome measures.

**Results:**

One thousand three hundred and sixty-six patients underwent mitral valve repair; 849 (62.2%) males and 517 (37.8%) females. Ages ranged from 18 to 90 years, with a mean age of 64 years. The overall 30-day mortality was 3.1% (43 patients). Among the 12 identified risk factors associated with increased mortality on univariate analysis, pre-operative hematocrit level was the only variable significantly correlated with mortality after undergoing multivariate analysis. 259 patients (19.1%) were discharged to a location other than home, an outcome associated with 22 identified risk factors. Among these risk factors, female gender, age, dialysis, pre-operative serum sodium, pre-operative serum albumin, and partial or full living dependency remained statistically significant following multivariate analysis. 126 patients (9.2%) experienced unplanned readmission. This outcome was associated with five risk factors, of which only dyspnea upon mild exertion was significant on multivariate analysis. Reoperation occurred in 105 patients (7.7%). Of the seven identified variables associated with reoperation, patient age, pre-operative platelet count, dyspnea upon mild exertion were independent predictors on multivariate analysis. 53 patients (3.9%) underwent reintubation, which was associated with 11 identified risk factors. Among them, patient age and pre-operative INR value were predictive of reintubation on multivariate analysis. 26 patients (1.9%) experienced stroke, of whom age was the only associated risk factor on both univariate and multivariate analysis. 31 patients (2.3%) experienced acute renal failure, which correlated with 11 risk factors on univariate analysis. Of these, only patient age and pre-operative hematocrit were identified as independent predictors on multivariate analysis.

**Conclusions:**

Outcomes are good following mitral valve repair. Although a substantial number of risk factors were found to be associated with adverse outcomes, only a small subset remained statistically significant following multivariate analysis. Identification of these risk factors may help guide clinical decision making with respect to which patients are the best candidates to undergo mitral valve repair.

## Highlights

• There is minimal literature on adverse 30-day outcomes following mitral valve repair.
• This study identified independent predictors of mortality, discharge location, readmission, reoperation, and reintubation.
• Management of these risk factors may help guide clinical decision making.

## Teaser

**Article Summary**The literature is limited regarding predictors of adverse 30-day outcomes following mitral valve repair. Through the use of the ACS NSQIP database, this study identified independent predictors of mortality, discharge location, readmission, reoperation, and reintubation. Management of the identified patient risk factors may help guide clinical decision making prior to undergoing surgery.

## 1 Introduction

Mitral regurgitation is the most common valvular disease within the United States [1]. For patients with mitral regurgitation, several treatment options currently exist including medical management, mitral valve replacement (via mechanical valve or bioprosthetic valve), and mitral valve repair (via percutaneous access, robotically assisted minimally invasive operative approach, or via open median sternotomy). Mitral valve repair has been considered the gold standard for those suffering from degenerative mitral valve disease as this procedure is associated with lower operative mortality, better long-term survival, and fewer valve-related complications when compared to prosthetic mitral valve replacement [2]. Further, surgical mitral valve repair, as opposed to percutaneous mitral valve repair, has been shown to be more effective in reducing the severity of mitral regurgitation [3].

Despite mitral valve repair's longstanding status as the superior treatment option for degenerative mitral valve disease, data on the risk stratification of patients undergoing mitral valve repair surgery are limited, with little known regarding the predictors of 30-day mortality and major morbidity. Much of the current literature is limited by factors such as the number of cases available for analysis, the number of preoperative potential predictors considered for analysis, and the fact that clinical data often comes from a single institution with only a few select surgeons performing the procedure.

Through the utilization of the American College of Surgeons National Surgical Quality Improvement Program (ACS NSQIP) database, data pertaining specifically to patients having undergone mitral valve repair can be extracted and analyzed to identify predictors of 30-day morbidity and mortality. The purpose of this study was to identify preoperative patient risk factors associated with morbidity and mortality within 30 days of mitral valve repair.

## 2 Methods

### 2.1 Data source

The study protocol was reviewed by the Institutional Review Board of the Icahn School of Medicine at Mount Sinai and determined to be exempt. All work was ensured to be fully compliant with the STROCCS criteria [4]. The Research Registry Unique Identifying Number is researchregistry5010. Data were derived from the Participant User File (PUF) of the ACS NSQIP for a retrospective analysis of patients having undergone mitral valve repair from 2011 to 2017. These data include any patient for which mitral valve repair was the principal operative procedure, which is defined as the most complex of all the procedures performed by the primary operating team during the trip to the operating room. Some patients within the dataset also underwent “other” or “concurrent” procedures, which are defined as additional surgical procedures performed by the same surgical team, or a different surgical team, under the same anesthetic which have CPT codes different from that of the principal operative procedure. The ACS NSQIP is a well validated quality improvement database that contains both preoperative and postoperative data for a wide variety of surgical procedures. The participant use date file (PUF) collected by NSQIP is de-identified and Health Insurance Portability and Accountability Act (HIPAA)-compliant, providing demographic, medical history, laboratory values, and perioperative data points for clinical cases submitted by over 600 participating sites across the United States for 30 days post procedure or until discharge. The data are collected prospectively at each site by a trained surgical clinical reviewer and monitored by ACS NSQIP to ensure data accrual and sampling methodologies are accurate.

Extracted cases from the PUF with missing information were excluded from the analysis. Further, only those variables that were present within the database from 2011 to 2017 were included within the analysis. Any variables that were removed or introduced during this timeframe were excluded.

The American College of Surgeons National Surgical Quality Improvement Program and the hospitals participating in the ACS NSQIP are the source of the data used herein; they have not verified and are not responsible for the statistical validity of the data analysis or the conclusions derived by the authors.

### 2.2 Variable definition

Potential predictors encompassed a variety of demographic, pre-operative, intra-operative, and post-operative variables. These variable included patient age, sex, race, height in inches, weight in lbs., transfer status (transferred either from the patient's home or a location other than their home), smoking status within one year, dyspnea, functional health status prior to surgery (independent, partially dependent, totally dependent), ventilator dependence, history of severe COPD (defined as patients with one or more of the following: functional disability from COPD including dyspnea or inability to perform activities of daily living, hospitalization in the past for treatment of COPD, chronic bronchodilator therapy requirement with oral or inhaled agents, an FEV1 of <75% of predicted on pulmonary function testing), diabetes mellitus with oral agents or insulin, ascites, congestive heart failure (CHF) within 30 days prior to surgery, hypertension requiring medication, disseminated cancer, open wound with or without infection and associated wound classification, steroid use for chronic condition, >10% loss of body weight in the last 6 months, bleeding disorders, pre-operative transfusion of ≥1 unit of whole/packed RBCs within 72 h prior to surgery, elective vs. emergency surgery, ASA classification, acute renal failure (pre-op), dialysis (pre-op), systemic sepsis (pre-op), pneumonia (pre-op), urinary tract infection (pre-op), and total operation time.

Preoperative lab values such as serum sodium, blood urea nitrogen, serum creatinine, serum albumin, total bilirubin, serum glutamic-oxaloacetic transaminase, alkaline phosphatase, white blood cell count, hematocrit, platelet count, partial thromboplastic time, prothrombin time, and International Normalized Ratio (INR) of prothrombin time values were also analyzed.

Potential postoperative predictors included length of total hospital stay, presence of surgical site infections, wound disruption, pneumonia, pulmonary embolism, requiring ventilator >48 h, progressive renal insufficiency, acute renal failure, urinary tract infection, stroke/CVA, cardiac arrest requiring CPR, myocardial infarction, bleeding transfusions (RBC within the first 72 h of surgery start time, DVT/thrombophlebitis, sepsis, septic shock, days from operation to discharge, and still in hospital > 30 Days. Postoperative 30-day outcome measures of interest included mortality, discharge destination, readmission, reoperation, reintubation, stroke, and acute renal failure.

### 2.3 Statistical analysis

Bivariate differences between perioperative risk factors and the identified outcomes of interest were analyzed using Student's t-test for continuous variables and chi-squared test for categorical variables. All variables that yielded a p-value < 0.05 were subsequently assessed via binary logistic regression to identify which variables, if any, were predictors of mortality, discharge destination, readmission, reoperation, reintubation, stroke, and acute renal failure. SPSS Statistics version 23.0 (IBM Corp, Armonk, NY) was utilized for all computation and statistical analysis.

## 3 Results

One thousand three hundred and sixty-six who underwent mitral valve repair from 2011 to 2017 were identified from the ACS NSQIP database. Patients included 849 (62.2%) males and 517 (37.8%) females. Ages ranged from 18 to 90 years, with a mean age of 64 years (Table 1).

**Table 1:** Patient characteristics.

Variables (Qualitative)	n (N = 1366)	Percent
*Sex*		
Female	517	37.8
Male	849	62.2
*Race*		
American Indian or Alaska Native	6	0.4
Asian	35	2.6
Black or African American	95	7.0
Native Hawaiian or Pacific Islander	3	0.2
White	1013	74.2
Elective Surgery	1095	80.2
*Comorbid conditions*		
Diabetes mellitus with oral agents or insulin	80	5.9
Current smoker within 1 year	183	13.4
History of severe COPD	106	7.8
Congestive heart failure in 30 days before surgery	304	22.3
Hypertension requiring medication	906	66.3
On dialysis	22	1.6
Steroid use for chronic condition	34	2.5
Bleeding disorders	111	8.1
Dysnpea on mild exertion	616	45.1
Partially or fully financially dependent	25	1.8
Transfer from a location other than home	132	9.7
**Variables (Quantitative)**	**Mean**	**Standard deviation**
Age	64.08	12.91
*Laboratory values*		
Pre-operative serum sodium (mEq/L)	138.72	3.10
Pre-operative BUN (mg/dL)	20.04	10.00
Pre-operative serum creatinine (mg/dL)	1.11	0.83
Pre-operative serum albumin (g/dL)	3.93	0.55
Pre-operative total bilirubin (mg/dL)	0.78	0.64
Pre-operative SGOT (units/L)	27.03	19.69
Pre-operative alkaline phosphatase (IU/L)	75.02	30.68
Pre-operative WBC ( × 10^9^/L)	6.99	2.25
Pre-operative hematocrit (%)	39.56	5.25
Pre-operative platelet count ( × 10^9^/L)	209.87	65.82
Pre-operative PTT (seconds)	32.96	10.98
Pre-operative INR of PT values	1.10	0.24

Associated comorbid conditions and mean preoperative lab values are also listed in Table 1. Table 2 demonstrates the number of cases associated with each of the primary outcome measures of interest. Overall 30-day mortality was 3.1% (43 patients). Discharge to a location other than home occurred in 259 patients (19.1%). Unplanned readmission occurred in 126 patients (9.2%). Reoperation occurred in 105 patients (7.7%). Reintubation occurred in 53 patients (3.9%). Stroke occurred in 26 patients (1.9%). Acute renal failure occurred in 31 patients (2.3%).

**Table 2:** 30-Day outcomes.

Outcome Description	N with outcome (%)	N without outcome (%)
Mortality	43 (3.1)	1323 (96.9)
Discharged to a place other than home	259 (19.1)	1099 (80.9)
Unplanned Readmission	126 (9.2)	1240 (90.8)
Reoperation	105 (7.7)	1255 (92.3)
Reintubation	53 (3.9)	1313 (96.1)
Stroke	26 (1.9)	1340 (98.1)
Acute Renal Failure	31 (2.3)	1335 (97.7)

On initial univariate analysis (Table 3), 12 variables were found to be associated with overall 30-day mortality including patient age (p = 0.002), non-elective surgery (p = 0.001), diabetes mellitus (p = 0.047), history of severe COPD (p = 0.001), CHF in 30 days before surgery (p = 0.017), bleeding disorders (p = 0.008), and several pre-operative lab values (p < 0.04 each). Discharge to a location other than home was associated with 22 identified risk factors on univariate analysis such as female gender (p < 0.001), age (p < 0.001), non-elective surgery (p < 0.001), diabetes mellitus (p < 0.001), history of severe COPD (p < 0.001), CHF in 30 days before surgery (p < 0.001), hypertension requiring medication (p < 0.001), actively on dialysis (p < 0.001), steroid use for chronic condition (p = 0.003), bleeding disorders (p < 0.001), partial or full living dependency (p = 0.014), transfer from a location other than home (p < 0.001), and 10 different pre-operative lab values (p < 0.02 each). Five risk factors were associated with unplanned readmission, including history of severe COPD (p = 0.030), CHF in 30 days before surgery (p = 0.004), dyspnea upon mild exertion (p = 0.008), as well as pre-operative serum albumin (p = 0.012) and hematocrit (p = 0.024). Reoperation was correlated with patient age (p < 0.001), hypertension requiring medication (p = 0.004), dyspnea upon mild exertion (p = 0.006), and pre-operative labs including WBC (p = 0.026), hematocrit (p = 0.019), platelet count (p = 0.014), and INR (p = 0.012). Eleven variables were associated with reintubation including patient age (p = 0.002), non-elective surgery (p = 0.003), history of severe COPD (p = 0.024), CHF in 30 days before surgery (p = 0.002), hypertension requiring medication (p = 0.042), steroid use for chronic condition (p = 0.049), and 5 pre-operative lab values (p < 0.02 each). Stroke was associated only with age (p = 0.019). Acute renal failure was associated with 11 risk factors including age (p = 0.001), history of severe COPD (p = 0.008), CHF in 30 days before surgery (p < 0.001), bleeding disorders (p = 0.002), and 7 pre-operative lab values (p < 0.04 each).

**Table 3:** Independent predictors of outcomes following univariate and multivariate analysis.

Outcome	Independent Risk Factor	N (%) or Mean Value with outcome	N (%) or Mean Value without outcome	Univariate p-value	Multivariate p-value (CI)
Mortality	Non-elective surgery	16 (1.2)	1350 (98.8)	p = 0.001	p = 0.868
	Diabetes Mellitus	6 (0.4)	1360 (99.6)	p = 0.047	p = 0.670
	History of severe COPD	10 (0.7)	1356 (99.3)	p = 0.001	p = 0.099
	CHF in 30 days before surgery	16 (1.2)	1350 (98.8)	p = 0.017	p = 0.482
	Bleeding disorders	9 (0.7)	1357 (99.3)	p = 0.008	p = 0.222
	Age (years)	69.98	63.89	p = 0.002	p = 0.554
	Pre-operative BUN (mg/dL)	27.29	19.80	p = 0.002	p = 0.295
	Pre-operative serum albumin (g/dL)	3.55	3.95	p = 0.001	p = 0.655
	Pre-operative WBC ( × 10^9^/L)	8.32	6.94	p = 0.011	p = 0.877
	Pre-operative hematocrit (%)	35.80	39.68	p < 0.001	p = 0.017 (0.855, 0.985)
	Pre-operative PTT (seconds)	40.94	32.73	p = 0.033	p = 0.260
	Pre-operative INR	1.24	1.10	p = 0.002	p = 0.098
Discharge to a place other than home	Gender (Female)	135 (9.9)	1223 (90.1)	p < 0.001	p = 0.033 (1.037,2.393)
	Non-elective surgery	90 (6.6)	1268 (93.4)	p < 0.001	p = 0.404
	Diabetes Mellitus	27 (2.0)	1331 (98.0)	p = 0.001	p = 0.198
	History of severe COPD	39 (2.9)	1319 (97.1)	p < 0.001	p = 0.553
	CHF in 30 days before surgery	109 (8.0)	1249 (92.0)	p < 0.001	p = 0.105
	Hypertension requiring medication	210 (15.5)	1148 (84.5)	p < 0.001	p = 0.056
	On dialysis	14 (1.0)	1344 (99.0)	p < 0.001	p = 0.037 (0.006, 0.850)
	Steroid use for chronic condition	13 (2.9)	1345 (99.0)	p = 0.003	p = 0.192
	Bleeding disorders	39 (2.9)	1319 (97.1)	p < 0.001	p = 0.067
	Partial or full living dependency	10 (0.7)	1348 (99.3)	p = 0.014	p = 0.049 (1.004, 20.715)
	Transfer from a location other than home	53 (3.9)	1305 (96.1)	p < 0.001	p = 0.633
	Age (years)	70.90	62.50	p < 0.001	p < 0.001 (1.041, 1.082)
	Pre-operative serum sodium (mEq/L)	137.78	138.95	p < 0.001	p = 0.009 (0.867, 0.980)
	Pre-operative BUN (mg/dL)	24.32	19.02	p < 0.001	p = 0.970
	Pre-operative serum creatinine (mg/dL)	1.40	1.04	p < 0.001	p = 0.663
	Pre-operative serum albumin (g/dL)	3.55	4.03	p < 0.001	p < 0.001 (0.238, 0.574)
	Pre-operative SGOT (units/L)	30.62	26.11	p = 0.019	p = 0.899
	Pre-operative alkaline phosphatase (IU/L)	81.20	73.46	p = 0.003	p = 0.674
	Pre-operative WBC ( × 10^9^/L)	7.56	6.85	p < 0.001	p = 0.988
	Pre-operative hematocrit (%)	36.65	40.23	p < 0.001	p = 0.351
	Pre-operative PTT	36.54	32.09	p < 0.001	p = 0.369
	Pre-operative INR	1.15	1.09	p < 0.001	p = 0.151
Unplanned Readmission	History of severe COPD	16 (1.2)	1350 (98.8)	p = 0.030	p = 0.386
	CHF in 30 days before surgery	41 (3.0)	1325 (97.0)	p = 0.004	p = 0.136
	Dyspnea upon mild exertion	71 (5.2)	1295 (94.8)	p = 0.008	p = 0.005 (0.353, 0.830)
	Pre-operative serum albumin (g/dL)	3.80	3.95	p = 0.012	p = 0.245
	Pre-operative hematocrit (%)	38.54	39.66	p = 0.024	p = 0.365
Reoperation	Hypertension requiring medication	83 (6.1)	1277 (93.9)	p = 0.004	p = 0.061
	Dyspnea upon mild exertion	61 (4.5)	1299 (95.5)	p = 0.006	p = 0.019 (0.384, 0.917)
	Age	68.98	63.66	p < 0.001	p = 0.028 (1.002, 1.043)
	Pre-operative WBC ( × 10^9^/L)	7.46	6.95	p = 0.026	p = 0.079
	Pre-operative hematocrit (%)	38.41	39.66	p = 0.019	p = 0.125
	Pre-operative platelet count ( × 10^9^/L)	194.47	211.06	p = 0.014	p = 0.039 (0.993, 1.000)
	Pre-operative INR	1.17	1.10	p = 0.012	p = 0.054
Reintubation	Non-elective surgery	18 (1.3)	1348 (98.7)	p = 0.003	p = 0.851
	History of severe COPD	9 (0.7)	1357 (99.3)	p = 0.024	p = 0.638
	CHF in 30 days before surgery	21 (1.5)	1345 (98.5)	p = 0.002	p = 0.204
	Hypertension requiring medication	42 (3.1)	1324 (96.9)	p = 0.042	p = 0.912
	Steroid use for chronic condition	4 (0.3)	1362 (99.7)	p = 0.049	p = 0.121
	Age	69.53	63.86	p = 0.002	p = 0.032 (1.003, 1.074)
	Pre-operative serum albumin (g/dL)	3.60	3.95	p < 0.001	p = 0.143
	Pre-operative alkaline phosphatase (IU/L)	86.25	74.56	p = 0.013	p = 0.466
	Pre-operative hematocrit (%)	37.53	39.64	p = 0.004	p = 0.438
	Pre-operative PTT	38.56	32.74	p = 0.013	p = 0.794
	Pre-operative INR	1.26	1.10	p = 0.001	p = 0.008 (1.453, 12.610)
Stroke	Age (years)	69.96	64.01	p = 0.019	p = 0.001 (1.007,1.082)
Acute Renal Failure	Age (years)	71.81	63.94	p = 0.001	p = 0.030 (1.005, 1.113)
	History of severe COPD	7 (0.5)	1359 (99.5)	p = 0.008	p = 0.205
	CHF in 30 days before surgery	15 (1.1)	1351 (98.9)	p < 0.001	p = 0.795
	Bleeding disorders	8 (0.6)	1358 (99.4)	p = 0.002	p = 0.226
	Pre-operative BUN (mg/dL)	31.84	19.76	p < 0.001	p = 0.263
	Pre-operative serum creatinine (mg/dL)	2.09	1.08	p = 0.005	p = 0.255
	Pre-operative serum albumin (g/dL)	3.52	3.94	p < 0.001	p = 0.229
	Pre-operative alkaline phosphatase (IU/L)	91.35	74.63	p = 0.006	p = 0.221
	Pre-operative hematocrit (%)	34.80	39.67	p < 0.001	p = 0.007 (0.808, 0.968)
	Pre-operative PTT (seconds)	37.91	32.85	p = 0.023	p = 0.847
	Pre-operative INR	1.20	1.10	p = 0.038	p = 0.465

Abbreviations.

COPD: Chronic obstructive pulmonary disease.

BUN: Blood urea nitrogen.

SGOT: Aspartate transaminase.

WBC: White blood cell count.

PTT: Partial thromboplastin time.

CHF: Congestive heart failure.

INR: International normalized ratio.

Further analysis was completed to assess which of the identified variables associated with each outcome of interest would maintain a statistically significant correlation following binary logistic regression (Table 3). Regarding overall 30-day mortality, multivariate analysis revealed that pre-operative hematocrit level was the only significantly correlated variable (p = 0.017), with survivors and non-survivors having an average pre-operative hematocrit level of 39.7% and 35.8% respectively. Among the 22 risk factors associated with discharge to a location other than home, female gender (p = 0.033), age (p < 0.001), dialysis (p = 0.037), pre-operative serum sodium (0.009), and pre-operative serum albumin (p < 0.001) remained statistically significant following multivariate analysis. Those discharged home had a mean age of 62.5 years, a mean pre-operative serum sodium of 139 mEq/L and a mean pre-operative serum albumin of 4.0 g/dL, while those discharged to a place other than home had a mean age of 70.9 years, a mean pre-operative serum sodium of 138 mEq/L, and a mean pre-operative serum albumin of 3.6 g/dL. Unplanned readmission was significantly associated only with dyspnea upon mild exertion on multivariate analysis (p = 0.005). Regarding reoperation, patient age (p = 0.028), dyspnea upon mild exertion (p = 0.019), and pre-operative platelet count (p = 0.039) were independent predictors of outcomes on multivariate analysis. Those requiring reoperation had an average age of 69.0 years and pre-operative platelet count of 194,000 per microliter of blood vs. 63.7 years and 211,000 per microliter of blood among those not requiring reoperation. Predictors of reintubation on multivariate analysis were patient age (0.032) and pre-operative INR (p = 0.008). Patients requiring reintubation had an average age of 69.5 years and a pre-operative INR of 1.3, while those not requiring reintubation had an average age of 63.9 years and pre-operative INR of 1.1. Regarding stroke, age was the only statistically significant risk factor on multivariate analysis. The average age of those who suffered from stroke was 69.9 years vs. 64.1 years among those who did not. Among the risk factors identified for acute renal failure, only age and preoperative hematocrit remained significant on multivariate analysis. Those with acute renal failure had an average age and pre-operative hematocrit of 71.8 years and 34.8% vs. 63.9 years and 39.7% among those without acute renal failure.

## 4 Discussion

While the outcomes following mitral valve repair are generally good, postoperative complications do occur. These complications have the potential to dramatically impact patient quality of life and in severe cases, may result in mortality. Through the identification of perioperative risk factors correlated with poor patient outcomes, we may be able to better identify which patients are most likely to have successful outcomes following mitral valve repair as well as improve patient optimization prior to undergoing mitral valve repair. It is our understanding that this is the first study to use the ACS NSQIP database to assess perioperative patient risk factors associated with morbidity and mortality within 30 days of mitral valve repair.

There is extensive literature regarding mortality rates following mitral valve surgery. The overall 30-day mortality rate of 3.1% found in the present study is higher than that found in much of the existing literature. Lazam et al. reported an operative mortality, defined as death occurring within 30 days of surgery or during the same hospitalization, of 1.3% in a study of 1709 patients having undergone mitral valve repair [2]. Additionally, in-hospital mortality following mitral valve repair was reported to be 2.1% by the European Association for Cardiothoracic Surgery in a study of 3231 patients, 1.6% by the Society of Thoracic Surgeons in a study of 7293 patients, 2% by the Society for Cardiothoracic Surgery in Great Britain & Ireland in a study of 3283 patients, and 2% by the German Society Thoracic and Cardiovascular Surgery in a study of 3335 patients [5]. Studies that reported higher mortality rates generally had smaller sample sizes. For example, Heikkinen et al. reported a 30-day postoperative mortality rate of 6.7% in a study of 164 patients following mitral valve repair [6].

One potential explanation for this difference in mortality rate may lie in the levels of expertise among the various institutions that submit outcomes data to ACS NSQIP. In a retrospective review of outcomes for 13,614 patients having undergone mitral valve repair, Gammie et al. observed an overall 30-day mortality rate of 2.12%, which was further sub-divided into 3.08% among the lowest-volume institutions (1–35 procedures/year) and 1.11% among the highest-volume institutions (>140 procedures/year) [7]. Reasons as to why procedures performed at high-volume hospitals are associated with better outcomes may include physician and institutional experience, selective referral of lower risk patients, and improved process of care management [8].

Regarding discharge location following mitral valve repair, our study indicated that 259 patients were discharged to a location other than home (19.1%). Henry et al. reported that among patients 80 years old or greater who underwent cardiac valve surgery, 40.1% were discharged to a place other than home (*n* = 307) [9]. Engoren et al. reported that following cardiac surgery, 53% of septuagenarians (*n* = 103) and 79% of octogenarians (*n* = 103) were discharged to a place other than home [10]. While these studies do provide some context, they of course are not directly comparable to the present study as they do not solely reflect mitral valve repair and are restricted to elderly patients whereas our patient age ranged from 18 to 89 years, with a mean age of 64 years.

The unplanned readmission rate of 9.2% within the 30 days of surgery was consistent with previous reports. Acker reported that among 126 patients that underwent mitral valve repair, 15 patients were readmitted (11.9%) within 30 days of the operation and 6 of those patients were readmitted specifically for cardiovascular causes (4.8%) [11]. In a study of 6896 Medicare beneficiaries having undergone mitral valve repair, Vassileva and co-workers reported a 30 day all-cause readmission rate of 22.0%, with one fifth of those readmissions being related to heart failure [12]. This higher percentage can likely be explained by the older age of the study population.

Our reoperation rate within 30 days of mitral valve repair of 7.7% was markedly higher than that found in previous studies. Nardi reported a mitral valve reoperation rate of 2.3% (*n* = 256) with the timing of reoperation ranging from 5 months to 5 years post-op [13], David reported that the probability of reoperation on the mitral valve at 10, 15 and 20 years following mitral valve repair was 4.1% (95% CI, 3.0–5.6%), 5.1% (95% CI, 3.8–7.0%), and 5.9% (95% CI, 4.3–8.0%) respectively [14], and Gillinov et al. reported reoperation rates of 1.3% at 1 year, 3.1% at 5 years, and 7.1% at 10 years in study of 1072 patients having undergone mitral valve repair [15].

Reintubation occurred in 49 patients (3.9%), which was consistent with other reports. Beverly et al. reported that among 1149 patients having undergone mitral valve repair, 3.2% were reintubated within 30 days of surgery [16]. Other studies assessing reintubation rates often assessed cardiac surgery overall as opposed to mitral valve repair specifically. For instance, Shoji et al. and Abdul-Zahoor reported reintubation rates of 7.26% (*n* = 1640) and 3.82% (*n* = 1229), respectively, within the postoperative period following cardiac surgery [17,18].

Post-operative stroke was found to occur in 1.9% of patients following mitral valve repair. This outcome was nearly equivalent to that found in Russo's 2008 study, which reported a stroke rate of 1.5 ± 0.4% among 897 patients within the first 30 days following mitral valve repair [19].

The 2.3% incidence of acute renal failure found in the present study was fairly similar to those found in other reports. Rosner and Okusa reported that among patients undergoing valvular surgery, the incidence of acute renal failure was 2.8% and the rate of acute renal failure requiring dialysis was 1.7% [20]. Additionally, Landoni et al. found that the crude incidence of acute renal failure following mitral valve replacement was 8% while that of mitral valve repair was 0.7% (p < 0.001) [21].

The present study identifies several important risk factors associated with 30-day mortality, of which only pre-operative hematocrit level remained statistically significant following multivariate analysis. Heikinnen reported that on multivariable analysis, patient age, history of prior cardiac surgery, and NYHA functional class were significantly associated with an increased risk of 30-day mortality following mitral valve repair [6]. Crabtree found that in a study of 257 patients undergoing mitral valve repair (34 patients undergoing mitral valve repair alone and 223 patients undergoing mitral valve repair combined with 1 or more other cardiac procedures), only previous CABG was associated with operative mortality by multivariate analysis [22]. There have also been several studies that examine predictors of longer term mortality. Feldman reported that functional mitral regurgitation, chronic obstructive pulmonary disease, older age, diabetes, and peripheral artery disease were independent predictors of 5-year mortality. However, the study population included patients who underwent surgical mitral valve repair as well as patients who underwent percutaneous mitral valve repair [23]. After a 10 year follow up following mitral valve repair, Nardi, et al. reported that the only independent predictor of late all‐causes mortality was older age at operation (71 ± 10 years in deceased patients versus 62 ± 12 years in survivors) [13]. Although a lower level of pre-operative hematocrit has not previously been mentioned in the literature as a predictor of 30-day mortality, the results of the present study may warrant further exploration into the effects of optimizing patient blood management prior to mitral valve repair as this is a modifiable risk factor.

Of the 22 variables correlated with discharge to a location other than home, multivariate analysis revealed that female gender, patient age, dialysis, pre-operative serum sodium, and pre-operative serum albumin remained independent predictors of discharge location. Some of these findings overlapped with those of the previously mentioned study by Henry et al., which indicated that older age, unmarried (single, separated, divorced, or widowed) patients, and those with major complications (defined as renal failure with or without dialysis required, stroke, pneumonia, deep sternal wound infection, leg infection, septicemia, or reoperation for bleeding) were statistically more likely to be discharged to other facilities following valve surgery [9].

Regarding unplanned readmission within 30 days of mitral valve repair, dyspnea upon mild exertion was the only statistically significant risk factor on multivariate analysis. In the study by Vassileva, the presence as well as the severity of preoperative heart failure was predictive of higher postoperative readmission rates in the elderly [12]. Additionally, although not isolated specifically to mitral valve repair, Pack reported that among patients undergoing heart valve surgery (*n* = 38,352) the strongest predictors of 1-month and 3‐month readmission included transfused units of packed red blood cells, presence of end stage renal disease, type of valve surgery (isolated mitral valve or combination valve procedures had a 25% higher risk of readmission compared to isolated aortic valve procedures), emergency hospital admission, and hospital length of stay [24].

Among the variables associated with reoperation within 30 days of surgery, multivariate analysis showed that patient age, dyspnea upon mild exertion, and pre-operative platelet count were independent predictors of this outcome. While the literature is limited regarding predictors of reoperation within 30 days of mitral valve repair, David's study indicated that on long term follow-up (median of 10 years), independent predictors of reoperation included isolated anterior leaflet prolapse, degree of myxomatous degeneration, Duran ring annuloplasty, and duration of cardiopulmonary bypass [14].

Independent predictors of reintubation within 30 days of mitral valve repair included patient age and pre-operative INR on multivariate analysis. In the previously mentioned study by Beverly, authors found that among patients having undergone cardiac surgery, those with poor baseline functional status and comorbid pulmonary, renal, or cardiac disease were statistically more likely to be reintubated in the first 30 days following surgery [16]. Although this study cannot be directly compared to the present findings as it identifies predictors following several cardiac surgeries as opposed to solely mitral valve repair, the current literature is limited with respect to reintubation rates within 30 days of mitral valve repair.

The only independent predictor of stroke identified in the present study was patient age. In the previously mentioned report by Russo et al., the investigators found that age was a univariate predictor of ischemic stroke within 30 days of mitral regurgitation surgery. Furthermore, they observed that age was an independent predictor of ischemic stroke at 30–180 days post-op as well as >180 days post-op. They also determined that independent of age, female gender was weakly associated with ischemic stroke within the first 30 days after mitral valve surgery. Other long term (>30 days) independent predictors of stroke included hypertension, mechanical mitral valve replacement, atrial fibrillation at surgery or before the event, and left atrial dimension >50 mm. Mitral valve repair was independently predictive of less-frequent ischemic stroke (RR 0.5, 95% CI 0.3 to 0.8) [19] when compared with mitral valve replacement.

Regarding the variables that were found to correlate with acute renal failure, only patient age and pre-operative hematocrit remained statistically significant on multivariate analysis. Landoni also identified age as an independent predictor of acute renal failure following mitral valve surgery. Other independent risk factors from that study included diabetes, preoperative renal impairment, mitral valve replacement (as opposed to mitral valve repair), emergency operation, re-operation for bleeding, and low-output syndrome [21]. Although lower pre-operative hematocrit has not been identified in prior studies as a potential risk factor for poor post-surgical outcomes, it is worth highlighting the fact that this variable has been independently associated with both acute renal failure as well as 30-day mortality in the present study.

As a result of utilizing the ACS NSQIP database, there are several limitations to the present study. ACS NSQIP does not necessarily reflect the outcomes of all hospitals across the US as academic medical centers tend to be overrepresented within the database. Furthermore, compared to a database such as that of the Society of Thoracic Surgeons, ACS NSQIP samples only about 20% of cases as opposed to capturing all cases. Additionally, we were unable to control for differences between participating institutions such as institutional size, location, case volume, and level of surgical expertise. Further, data analysis pertains only to 30-day outcomes following mitral valve repair, excluding any conclusions that can be drawn relating to predictors of longer-term outcomes. There are also several potentially important patient factors absent from the database including anatomic and physiologic variability between patients, severity of mitral regurgitation/case complexity, severity of comorbidities, and patient surgical risk scores. ACS NSQIP lacks some of the pre-and post-operative risk factors frequently associated with cardiac surgery such as measures of left ventricular function, cardiac functional status, and arrhythmias. It also lacks some of the intra-operative risk factors that might impact morbidity and mortality such as repair technique, surgery duration, and duration of cardiopulmonary bypass or aortic cross clamping times.

## 5 Conclusion

Mitral valve repair generally leads to good outcomes for patients, but it is still critical to identify which patients might be at a higher risk of post-operative morbidity or mortality. This study offers some additional insight into the predictors of several adverse outcomes. While numerous patient variables were found to be associated with morbidity and mortality, few of the identified risk factors remained statistically significant on multivariate analysis. Knowledge of these predictors of adverse outcomes can help guide physicians regarding which patients are best suited to undergo mitral valve repair as well as how best to optimize those patients prior to surgery.

## Conflicts of interest

None.

## Sources of funding for your research

None.

## Ethical approval

Waiver was provided by the Institutional Review Board of the Icahn School of Medicine at Mount Sinai.

## Consent

Not applicable. All data used herein was previously de-identified.

## Author contribution

Study design: Reisman, Leitman.

Data Acquisition: Reisman, Thomas, Boateng.

Manuscript preparation: Reisman, Thomas, Boateng, Leitman.

Critical revision of manuscript: Reisman, Thomas, Boateng, Leitman.

Final approval of manuscript: Reisman, Thomas, Boateng, Leitman.

## Registration of research studies

1. Name of the registry: Research Registry
2. Unique Identifying number or registration ID: researchregistry5010
3. Hyperlink to the registration (must be publicly accessible): https://www.researchregistry.com/browse-the-registry#home/registrationdetails/5d2868036528ba0011374462/

## Guarantor

I. Michael Leitman, M.D.

## Provenance and peer review

Not commissioned, externally peer reviewed.

## Appendix A Supplementary data

strocss_statement_checklist strocss_statement_checklist
